# Differences in distribution of anterior segmental medullary arteries in the cervical and thoracolumbar spinal cord: the “inseln” were characteristics in the cervical spinal cord

**DOI:** 10.1007/s12565-019-00498-y

**Published:** 2019-08-09

**Authors:** Jun Kanazawa, Jun Yan, Jiro Hitomi

**Affiliations:** Department of Anatomy, School of Medicine, Iwate Medical University, 1-1-1 Idaidori, Yahaba-cho, Shiwa, Iwate, 028-3694 Japan

**Keywords:** Anterior segmental medullary artery, Anterior spinal artery, Cervical spinal cord, Insel, The artery of Adamkiewicz

## Abstract

Anterior segmental medullary arteries cause spinal cord infarction due to circulatory disturbance, but are difficult to identify in diagnostic images. This study investigated the arterial distribution from the cervical to lumbar segments of the dissecting spinal cord in 100 cadavers. The 488 arteries were distributed from C2 to L2, of which 252 arteries from C2 to C8 were slightly dominant on the right side, but 236 arteries from Th1 to L2 were obviously dominant on the left side. This change occurred at the C8 and Th1 segments. Of the 252 arteries, 37.30% (94 arteries) were divided into ascending and descending branches. Both branches formed loops by anastomosis with the anterior spinal arteries. The loops, called “insel”, have an unclear distribution and form. We focused on the features of inseln and found 63 of them in the cervical spinal cord of 45 cadavers. Their numbers and forms differed depending on whether the 94 arteries were bilateral (type A) or unilateral (type B), but the long axis of the insel was limited to one cervical segment. 90.63% of type A were bilateral at the same level, and 70% of type B were on the right side. The former always formed the insel. Further, 94 arteries were distributed from C2 to C7, 82.98% of which were concentrated at C3–C5. Therefore, the arterial blood supply of the spinal cord may differ between the cervical spinal cord and the thoracolumbar cord.

**Electronic supplementary material:**

The online version of this article (10.1007/s12565-019-00498-y) contains supplementary material, which is available to authorized users.

## Introduction

Most morphological studies on the blood vessels of the spinal cord are based on gross anatomy. In the blood vessels of the spinal cord, the distribution of the anterior segmental medullary artery (ASMA) has been extensively studied (Adamkiewicz 1882; Miyadi 1931). The spinal cord is supplied blood from the anterior and posterior spinal arteries, which are branches of the vertebral artery, and from the ASMA and posterior segmental medullary arteries of the spinal nerve (Marinlovic et al. 1997; Thron 1998). The anterior spinal artery (ASA) perfuses two-thirds of the ventral side, but the upper and lower continuities are poor. Therefore, it is nourished through the segmental spinal cord branches of the vertebral, ascending cervical, deep cervical, intercostal, lumbar, lateral and middle sacral arteries (Lasjaunias et al. 1990). Each segmental spinal artery becomes the ASMA. The ASMA accompany the anterior radicular until it reaches the surface of the spinal cord. In this case, the ASMA is basically anastomosed in a T shape to the ASA longitudinally running through the spinal cord (Frick et al. 2000). In addition, in the ASMA, the thickest artery is called the artery of Adamkiewicz (AKA) (Adamkiewicz 1882). The origins of the AKA are 83.9% from Th12 to L3 and 67.7% on the left side (Biglioli et al. 2000). The AKA and vein of Adamkiewicz are similar, so it is hard to distinguish in the diagnostic images (Yoshioka and Tanaka 2009).

The ASMA causes spinal cord infarction due to circulatory disturbance. This is a rare disease that occurs in 1–2% of stroke cases (Sandson and Friedman 1989). The etiology refers to aortic dissection, rupture of the aortic aneurysm, and surgery as the main causes, but the etiology in many cases have not been identified (Cheshire et al 1996; Novy et al. 2006; Cheng et al. 2008). The infarct lesion occurs more frequently in the thoracolumbar cord and rarely occurs in the cervical cord (Nedeltchev et al. 2004; Kameda et al. 2010; Teranishi et al. 2017). Therefore, this study aimed to investigate the distribution of the ASMA and AKA from the cervical spinal cord to the lumbar spinal cord in 100 cadavers. As a result, in the cervical spinal cord, we found a loop consisting of the ASMA and ASA. This is a loop in which the ASMA in front of the cervical spinal cord divides into ascending and descending branches and anastomoses to the ASA at two places. This loop is called the “insel” (Miyadi 1931; Kudo et al. 1983). Kadyi (1889) and Adachi (1928) investigated insel in the vertebral and basilar arteries, but the distribution and form have not been elucidated. The insel is difficult to identify in diagnostic images because it is thinner than the ASMA. Therefore, in addition to the distribution of the ASMA, we clarified the distribution and form of the inseln in the cervical spinal cord.

## Materials and methods

The spinal cords of 100 cadavers fixed in formalin solution were used. The spinal cords had no external abnormality, and none of the deaths were caused by spinal cord diseases. The cadavers were obtained from Iwate Medical University during anatomical practices from 2015 to 2018. The cadavers were all Japanese and included 55 males and 45 females. The ages of the deceased ranged from 50 to 102 years, with a mean of 80.4 years. Anatomy was performed according to the anatomy guidelines after approval of the Iwate Medical University Ethics Committee (Ethics no. H 27–103).

First, the spinal cord wrapped in the dura from the spinal canal was dissected. The spinal cord was cut between the medulla oblongata and the upper cervical spinal cord. Then, the spinal nerve root was cut, and the spinal cord was taken. Because the spinal nerve root was cut, the spinal branch was excluded from the ASMA.

**Fig. 1:**
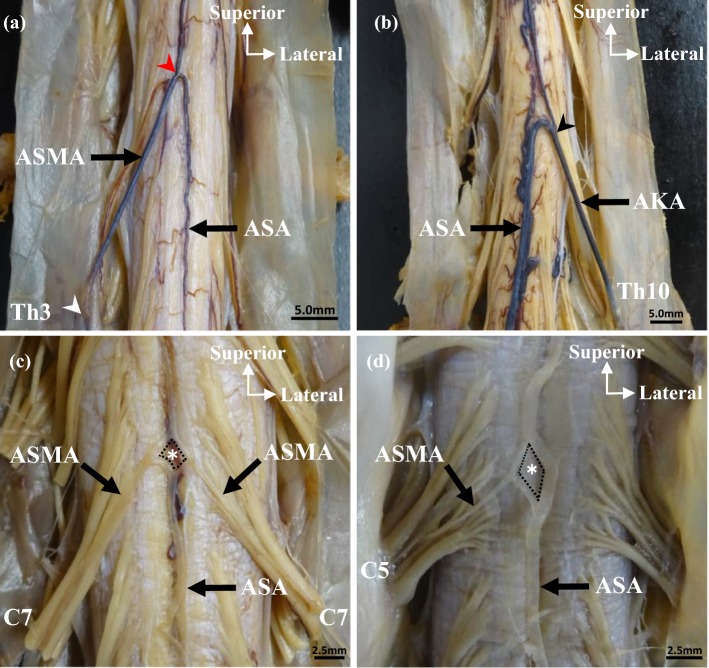
The artery of Adamkiewicz (*AKA*), anterior spinal artery (*ASA*), and anterior segmental medullary arteries (*ASMA*) of the spinal cord in 4 cadavers. **a***ASMA* in Th3. This length was measured from the point where *ASMA* penetrated the spinal dura mater (white arrowheads) to the confluence of the *ASA* (red arrowheads). **b***AKA* in Th10. The thickness was measured as the confluence (black arrowheads) of the *AKA* and *ASA*. **c** and** d** The inseln (asterisk) consisted of bilateral or unilateral *ASMA* and *ASA*. The dashed box indicates circumference of the insel. *AKA,* the artery of Adamkiewicz; *ASA,* anterior spinal artery; *ASMA,* anterior segmental medullary artery; *C5* and *C7,* 5th and 7th cervical segment; *Th3* and *Th10*, 3rd and 10th thoracic segments

Second, the ASMA and AKA were observed under macroscopy and stereoscopy. After identifying the level of the spinal nerve root, we investigated the number, level, side, and length of the ASMA from the cervical spinal cord to the lumbar spinal cord. This length was measured from the point where ASMA penetrated the spinal dura mater to the confluence of the ASA (Fig. 1a). Then, we investigated the number, level, side, and thickness of the AKA from the thoracic spinal cord to the lumbar spinal cord. The thickness was measured as the confluence of the AKA and ASA (Fig. 1b).

The bilateral or unilateral ASMA in front of the cervical spinal cord divides into the ascending and descending branches and anastomoses to the ASA at two places. The anastomosis of the ASA and ASMA forms a loop, known as insel (Fig. 1c, d). Finally, the insel in the cervical cord was observed under macroscopy and stereoscopy. The items of observation for the insel were as follows: (1) number and circumference, (2) form and long axis, and (3) segmental distribution of the ASMA. The minimum circumference to be ≥ 1.0 mm because of the possibility of overlooking with the naked eye. In addition, the insel was bounded by three or four sides. Thus, the circumferences were calculated by measuring the length of each side with a measurement device and then summing up the total length of these three or four sides. Measurements were performed using 6 in./150 mm electronic digital calipers (least count = 1/100 mm).

## Results

### Distribution of the ASMA and AKA

**Fig. 2:**
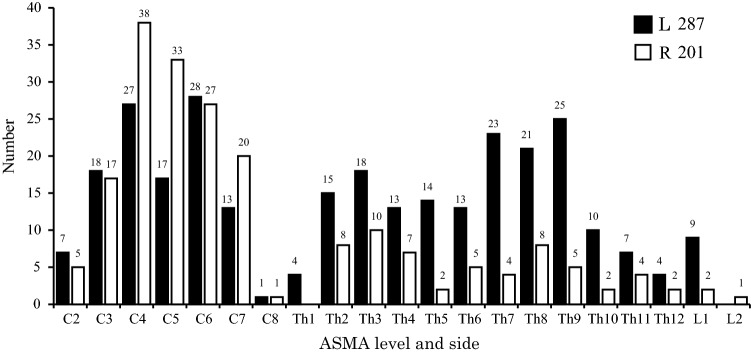
Level and side of the anterior segmental medullary arteries (ASMA; *n* = 488). A total of 252 ASMA of the cervical spinal code were right-side dominant, while 236 ASMA of the thoracolumbar cord were left-side dominant. The difference between left and right changes between C8 and Th1. *L*, left; *R*, right

We studied 488 ASMA from the spinal cords of 100 cadavers (Fig. 2). The distribution of the ASMA was as follows: 252 (51.6%) from C2 to C8, 224 (45.9%) from Th1 to Th12, and 12 (2.5%) from L1 to L2. In each spinal cord, the highest numbers of ASMA were found at C4, Th9, and L1. Of the 488 ASMA of the spinal cord, 287 (58.8%) were on the left side and 201 (41.2%) were on the right side. Of the 252 ASMA in the cervical spinal cord, 58 (11.9%) were bilateral at the same level, 82 (16.8%) were on the left side, and 112 (22.9%) were on the right side. Of the 224 ASMA in the thoracic spinal cord, 167 (34.2%) were on the left side and 57 (11.7%) were on the right side. Of the 12 ASMA in the lumbar spinal cord, 9 (1.9%) were on the left side and 3 (0.6%) were on the right side. The ASMA in the cervical spinal cord slightly dominant on the right side and the bilateral ASMA remained particularly in the cervical spinal cord in contrast to the thoracolumbar spinal cord. Of the ASMA, 80.7% were anastomosed to the ASA in a T shape. The length of the ASMA ranged from 4.0 to 93.5 mm, with a mean of 49.7 mm.

In addition, we studied the number of ASMA in the 100 individual cadavers. The ASMA ranged from 2 to 10 with a mean of 4.9 ASMA per spinal cord. The most common number ASMA was 4 seen in 24 cases, followed by 5 ASMA in 19. In the 100 cervical spinal cords, the ASMA ranged from 0 to 6 with a mean of 2.5 ASMA (left 1.1 ASMA, right 1.4) per spinal cord, whereas in the 100 thoracic spinal cords, the ASMA ranged from 0 to 5 with a mean of 2.3 ASMA (left 1.7 ASMA, right 0.6) per spinal cord. In both the cervical and thoracic spinal cords, the most common number of ASMA was 2, seen in 34 and 53 cases, respectively. In the 100 lumbar spinal cords, the ASMA ranged from 0 to 2 with a mean of 0.1 ASMA (left 0.1 ASMA, right 0) per spinal cord. For the lumbar spinal cord, 89 cases had 0 ASMA. In a single spinal cord, the number of ASMA in the cervical spinal cord was, thus, the greater than it in the thoracolumbar spinal cord.

**Fig. 3:**
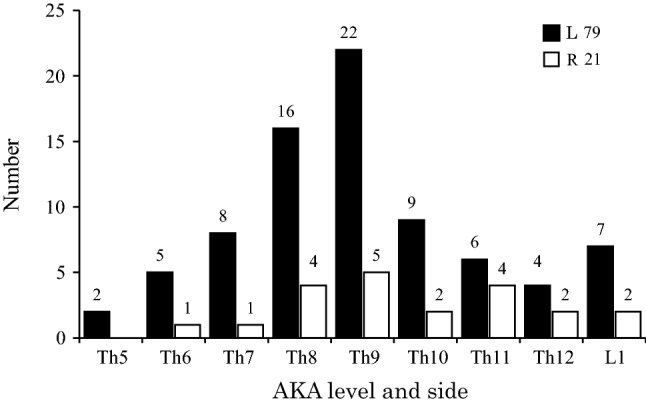
Level and side of the artery of Adamkiewicz (AKA; *n* = 100). A total of 100 AKA of the thoracolumbar cord were left-side dominant, while Th9 had the highest number of AKA. AKA ranges from Th5 to L1. *L*, left; *R*, right

In addition, we studied 100 AKA from the spinal cords of 100 cadavers (Fig. 3). 91 AKA were distributed from Th5 to Th12, and 9 AKA were distributed only at L1. Of the AKA, 83% were observed from Th8 to L1. In the spinal cord, Th9 had the highest number of AKA. Of the 100 AKA of the spinal cord, 79 were on the left side and 21 were on the right side. Of the 91 AKA in the thoracic cord, 72 were on the left side and 19 were on the right side. Of the 9 AKA of the lumbar cord, 7 were on the left side and 2 was on the right side. The thicknesses of the AKA ranged from 0.60 to 1.21 mm, with a mean of 0.91 mm. In 2 cases, the AKA was not the lowest artery. When the AKA was at Th9 or Th5 on the left side, the lowest artery was at L2 on the right side or L1 on the left side.

### Distribution and form of the insel

#### Number and circumference

We studied 63 inseln from the cervical spinal cords of 45 cadavers, of which 28 had 1 insel, 16 had 2 inseln, and 1 had 3 inseln. The circumference of the inseln ranged from 3.4 to 68.0 mm, with a mean of 17.1 mm. The ASMA in the cervical spinal cord forming the insel was bilateral or unilateral. The former was classified as type A, and the latter was classified as type B. Furthermore, type A was further classified as types A1 (same level) and A2 (not the same level); and type B as types B1 (right side) and B2 (left side). Of the 63 inseln, 33 (52.38%) were type A and 30 (47.62%) were type B. 30 inseln (47.62%) were type A1; 3 (4.76%) were type A2; 21 (33.33%) were type B1; and 9 (14.29%) were type B2 (Table 1).

**Table 1:** Number, form, and long axis of the inseln consisting of the anterior segmental medullary arteries and the anterior spinal artery

Inseln (*N* 63)	Number	Form			Long axis		
Type	[*N* (%)]	Rhombus [*N* (%)]	Triangle [*N* (%)]	Oblong [*N* (%)]	One cervical segment [*N* (%)]	Two cervical segments [*N* (%)]	Three cervical segments [*N* (%)]
A 1 (same level)	30 (47.62)	25 (39.68)	2 (3.18)	3 (4.76)	28 (44.45)	2 (3.18)	
A 2 (not the same level)	3 (4.76)			3 (4.76)		2 (3.18)	1 (1.59)
B 1 (right side)	21 (33.33)	2 (3.17)	18 (28.57)	1 (1.59)	20 (31.75)		1 (1.59)
B 2 (left side)	9 (14.29)	1 (1.59)	7 (11.11)	1 (1.59)	8 (12.70)		1 (1.59)
Total (inseln)	63 (100)	28 (44.44)	27 (42.86)	8 (12.70)	56 (88.90)	4 (6.36)	3 (4.77)

A, bilateral anterior segmental medullary artery; B, unilateral anterior segmental medullary artery

#### Form and long axis

**Fig. 4:**
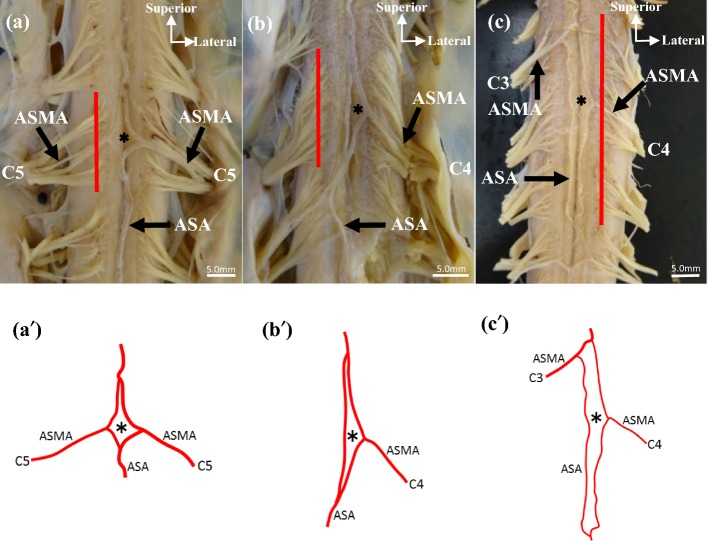
Form and long axis of the inseln in front of the cervical spinal cord. **a** Type A1, the form is a rhombus (asterisk) and the long axis limits to one cervical segment (red line). **b** Type B2, the form is a triangle (asterisk) and the long axis is the same as in **a** (red line). **c** Type A2, the form is an oblong (asterisk) and the long axis limits to three cervical segments (red line). **a´, b´, c´** Illustration is the form (asterisk) shown in **a, b, c**. *ASA,* anterior spinal artery; *ASMA,* anterior segmental medullary artery; *C3–C5,* 3rd to 5th cervical segment

The forms of the inseln were rhombus, triangle, and oblong (Fig. 4). The insel is described as a rhombus if it has four sides and its long axis is one cervical segment. It is described as a triangle if has three sides and its long axis is one cervical segment. It is described as oblong if it has three or four sides and its long axis is two or three cervical segments. Rhombic and oblong inseln were located on the fissura mediana, and 66.7% of those with a triangular form were on the right side of the ASA. The forms of type A were rhombus (39.68%), oblong (9.52%), and triangle (3.18%), and those of type B were triangle (39.68%), rhombus (4.76%), and oblong (3.18%), respectively. The forms of type A1 were rhombus (39.68%), oblong (4.76%), and triangle (3.18%), respectively, and that of type A2 was oblong (4.76%). The type B1 were triangle (28.57%), rhombus (3.17%), and oblong (1.59%), and the type B2 were triangle (11.11%), oblong (1.59%), and rhombus (1.59%), respectively (Table 1). Most of the inseln were rhombus and triangle, and only few were oblong.

The long axis of the insel was limited between one and three cervical segments (Fig. 4). 56 inseln (88.90%) were limited to one cervical segment. 4 inseln (6.36%) and 3 inseln (4.77%) were limited to two and three cervical segments, respectively (Table 1). In 88.90% of the inseln, types A1 and B had the same frequencies. In addition, type A1 (44.45%) corresponded to rhombic and triangular inseln (42.86%) and partly to oblong inseln (1.59%). Type B (44.45%) consisted of rhombic and triangular inseln. In 6.36% of ASMA, types A1 and A2 had the same frequency. Additionally, all of 4 (6.36%) and 3 inseln (4.77%) corresponded to oblong inseln.

#### Segmental distribution of ASMA

Of the 252 ASMA in the cervical spinal cord, 37.30% (94 arteries) were involved in the formation of inseln (Table 2). The 236 ASMA in the thoracolumbar cord were not involved in the formation of inseln.

**Table 2:** Number and segmental distribution of the anterior segmental medullary arteries in the cervical spinal cord involved in the formation of inseln

Arteries type (*N* 94)	Number	Segmental distribution					
	[*N* (%)]	C2 [*N* (%)]	C3 [*N* (%)]	C4 [*N* (%)]	C5 [*N* (%)]	C6 [*N* (%)]	C7 [*N* (%)]
A 1 (same level)	58 (61.70)		14 (14.90)	18 (19.15)	18 (19.15)	6 (6.38)	2 (2.13)
A 2 (not the same level)	6 (6.38)		1 (1.06)	3 (3.19)	2(2.13)		
B 1 (right side)	21 (22.34)	1 (1.06)	2 (2.13)	6 (6.38)	7 (7.45)	3 (3.19)	2 (2.13)
B 2 (left side)	9 (9.57)			5 (5.32)	2 (2.13)		2 (2.13)
Total (arteries)	94 (100)	1 (1.06)	17 (18.09)	32 (34.04)	29 (30.86)	9 (9.57)	6 (6.39)

A, bilateral anterior segmental medullary artery; B, unilateral anterior segmental medullary artery; C2–7, 2nd to 7th cervical segment

Of the 94 arteries, 68.08% (64 arteries) were type A and 31.91% (30 arteries) were type B. Types A1 and A2 accounted for 61.70% (58 arteries) and 6.38% (6 arteries) of the ASMA, respectively. Types B1 and B2 were found in 22.34% (21 arteries) and 9.57% (9 arteries) of the ASMA. Type A1 matched the number of bilateral arteries in the cervical spinal cord. 94 arteries were distributed from C2 to C7, and occupied C4 (32 arteries) and C5 (29 arteries). Type A1 was distributed from C3 to C7, with high frequencies at C4 and C5. Type A2 were found at C3 (an artery) and C5 (2 arteries) on the right and, C4 on the left (3 arteries). The 3 cases of type A2 in Table 1 consisted of 1 case at C3 on the right and C4 on the left, and 2 cases at C5 on the right and C4 on the left. Type B1 was distributed at all levels.

## Discussion

The number of ASMA ranged from 3 to 10, with a mean of 5.9 ASMA per spinal cord, which are dominant on the left side (Miyadi 1931). Therefore, the mean and difference between the left and right are similar. However, the arteries of the cervical spinal cord were dominant on the right side as compared with the thoracolumbar cord. This is due to the fact that the ASMA of the cervical spinal cord enters bilaterally at the same level, there is little difference between the left and the right (C2, C3, C6, and C8), and 67.16% of the ASMA occupy the right side from C3 to C7. Moreover, 5 anomalous cases of the aortic arch were found. 4 cases belonged to type C of Adachi (1928), and 1 case belonged to the right aortic arch (Yan et al. 2016). Normally, the diameter of the vertebral artery is thicker on the left side, but it was thicker on the right side in the case of type C and right aortic arch. In fact, the number of ASMA on the right side in the 4 cases was double that on the left side. As the cervical spinal cord depends on the vertebral artery (Dommisse 1975), we think that the vertebral artery influences it. Mannen (1981) reported that the ASMA in the cervical cord enters at C3, C5, C6, or C7 and it in the lumbar cord enters at L1 or L2. In addition, he states that many cases have two ASMA in the thoracic cord; one of these enters at Th3 or Th4, while the other enters at Th8 or Th9. But in our cases, it entered from C4, C6, Th3, Th8, Th9, and L1. Individual differences in numbers were observed, and the levels are not always constant but it concentrated in the middle and lower cervical spinal cord (C3–C7). Jellinger (1996) reported 2 or 3 ASMAs in the thoracic spinal cord. Therefore, we think that there may be one ASMA in the upper thoracic spinal cord from Th1 to Th4 and one at either Th8 or Th9. As the number of ASMA in the lumbar spinal cords is clearly less than those of the others, it may not exist. In addition Kudo et al. (1983) did not observe arteries entering from C1, so the arteries are important from C2 or lower.

Of the AKA, 83% were distributed from Th8 to L1. The distribution is wider than the result reported by Biglioli et al. (2000), but not contradictory to the concentration from the lower thoracic spinal cord to the upper lumbar spinal cord. Many cases (27%) entering Th9 were consistent with the results of Miyadi (1931). In all the cases, AKA was found to have a hairpin-like curve when anastomosing into the ASA (Yoshioka and Tanaka 2009). In addition, it tends to become thinner in the periphery. Of the arteries, 79% originated from the left side because 99 cases of thoracic aorta were on the left side. The thickness of the AKA ranged from 0.60 to 1.21 mm, with a mean of 0.91 mm. This is almost the same as the mean (0.87 mm) reported by Suh and Alexander (1939). None of the cases had the same thickness, but in 2 cases, the AKA was not the lowest artery. As confirmed by Goto and Shiraishi (1988) in 1 case, AKA is not always the lowest artery.

All the inseln were found in the 45 cervical spinal cords. As the mean circumference was 17.1 mm, the arteries were difficult to identify on the diagnostic images. No insel in 55 cervical spinal cords and no bilateral ASMA were found. The number of bilateral arteries in 100 spinal cords corresponded with the number of type A1 ASMA in Table 2. Therefore, when bilateral ASMA were found at the same level, the insel was always formed. Under the same conditions, the formation of the insel is likely to happen rather than being anastomosed into a T shape, to preserve the continuity of the ASA. In the case of type B, despite the fact that the number of arteries was 31.91%, the same number of type A1 was found. As most of the type B were type B1, it can be concluded that the dominance of the 252 arteries of the cervical spinal cord on the right side did have an effect on this feature.

The forms of the inseln were rhombus and triangle. The rhombic inseln tended to be of type A, especially type A1. In addition, triangular inseln tended to consist of type B, especially type B1. Therefore, the frequency of oblong inseln was as low as 12.70%, but these inseln consisted of type A when they were formed. Of the inseln, 88.90% were observed in one cervical segment. As types A1 and B had the same frequencies, we think that the long axis of the insel is limited to one cervical segment regardless of the type. In addition, the frequency of inseln of two and three cervical segments was as low as 11.13%. Therefore, the blood supply of the cervical spinal cord could be sufficient because the insel was limited to one cervical segment, or ASMA was anastomosed to the ASA in a T shape without expanding the range.

The long axis of the insel is limited to one cervical segment, but anastomosis increases due to the change in form. This is because 19.26% of the ASMA do not anastomose to the ASA in a T shape but divide into ascending and descending branches and anastomose at two places. Although the incidence of insel is low, collateral circulation is formed when admitted. Unlike the case of the thoracolumbar cord, spinal cord infarction rarely occurs in the cervical spinal cord. This fact may be influencing in the collateral circulation of insel. Infarction with respiratory failure may also be found in the cervical spinal cord (Howard et al. 1998).

The middle thoracic cord has a large distance between the upper and lower arteries. In addition, compared with the cervical and lumbar intumescence, blood vessels are thin, which are likely to become ischemic (Watanabe and Okubo 2008). Arteries forming inseln were distributed from C2 to C7, and the formation rate of C3–C5 was as high as 82.98%. This corresponds to 30.95% of the 252 ASMA. Therefore, inseln tend to form into segments where the arteries concentrate. However, the insel formation rate at C6 and C7 was as low as 15.96%, and that at C8 was nil. In addition, the insel between two and three segments did not expand to the thoracic spinal cord. Therefore, the collateral circulation of the insel does not supplement blood supply to the thoracic spinal cord. By contrast, 37% of the AKA appeared in the middle thoracic spinal cord from Th5 to Th8, which we supposed was to supplement the blood supply. Like the thoracic spinal cord, the lumbar spinal cord had no insel because only 2.46% of the 488 ASMA were present. As 63% of the AKA are distributed from Th9 to L1, the supplementation of blood supply to the lumbar spinal cord is also sufficient. Thus, the AKA is involved in the blood supply to the thoracolumbar spinal cord. However, the collateral circulation of the insel is not involved in the thoracolumbar spinal cord and was limited to the cervical spinal cord. Therefore, the possibility that the arterial blood supply differs from the thoracolumbar cord is highly likely. If it plays a role in collateral circulation, the operation of the spinal cord blood vessel requires the recognition of the insel to prevent bleeding. The inseln are considered to be in the process of proceeding to the bilateral ASMA to a single ASA (Hiura 1978). By further clarifying the distribution and form of the insel, it may be useful for inferring the arterial formation process in the cervical spinal cord.

## Conclusions

The numbers and forms of the 63 inseln found in 45 cervical spinal cords differ depending on whether the ASMA was type A (bilateral) or B (unilateral), but the long axis of the insel was limited to one cervical segment. The 94 ASMA of the cervical spinal cord tended to form the insel when the ASMA was type A1 (bilateral at the same level) or B1 (on the right side). Our results suggest that the insel consisted of the ASMA in the cervical spinal cord, especially from C3 to C5. Therefore, the arterial blood supply of the spinal cord may differ between the cervical spinal cord forming the insel and the thoracolumbar cord. Moreover, this study could serve to contribute information about the process of artery formation in the cervical spinal cord.

## Electronic supplementary material

Below is the link to the electronic supplementary material.

Supplementary file1 (PPTX 104 kb)

## References

[CR1] Adachi B (1928). Das Arteriensystem der Japaner..

[CR2] Adamkiewicz A (1882). Die Blutgefässe des menschlichen Rückenmarkes. II. Die Gefässe der Rückenmarksoberfläche. Sitzungsberichte der Kaiserlichen Akademieder Wissenschaften. Mathematisch Naturwissenschaftliche Classe.

[CR3] Biglioli P, Spirito R, Roberto M, Grillo F, Cannata A, Parolari A (2000). The anterior spinal artery: the main arterial supply of the human spiral cord: a preliminary anatomic study. J Thorac Cardiovasc Surg.

[CR4] Cheng MY, Lyu RK, Chang YJ (2008). Spinal cord infarction in Chinese patients. Cerebrovasc Dis.

[CR5] Cheshire WP, Santos CC, Massey EW (1996). Spinal cord infarction: etiology and outcome. Neurology.

[CR6] Dommisse GF (1975) The Arteries and Veins of the Human Spinal Cord from Birth. Edinburgh:Churchill Livingstone:15–34

[CR7] Frick H, Leonhardt H, Starck D (2000) Human Anatomy 2. Nishimurasyoten :238–239

[CR8] Goto N, Shiraishi N (1988). Morphology of Arteria radicularis magna anterior et posterior. Jpn J Spine Spinal Cord.

[CR9] Hiura A (1978). Distribution of radicular arteries in the spinal cord. Fukushima J Med Sci.

[CR10] Howard RS, Thorpe J, Barker R, Revesz T, Hirsch N, Miller D, Williams AJ (1998). Respiratory insufficiency due to high anterior cervical cord infarction. J Neurol Neurosurg Psychiatry.

[CR11] Jellinger K (1996). Zur Orthologie und Pathologie der Ruckenmarksdurchblutung.

[CR12] Kadyi H (1889) Über die Blutgefäße des menschlichen Rückenmarkes. Lemberg: Gubryowicz und Schmidt:30

[CR13] Kameda T, Doi H, Kawamoto Y (2010). Clinical features and prognosis in 14 cases of spinal cord infarctions. Jpn J Stroke.

[CR14] Komiyama M (2015). Functional anatomy of the perforating arteries of the brain and spinal cord: from the viewpoint of segmental structures of the central nervous system. Jpn J Neurosurg Tokyo.

[CR15] Kudo O, Fukushi T, Kasai T (1983). Segmental distribution of *A. radicularis anterior* and *A. radicularis magna*. Arch Orthop Trauma Surg.

[CR16] Lasjauniasa P, Berenstein A, ter Brugge KG (1990). Surgical neuroangiography 1: spine and spinal cord arteries and veins.

[CR17] Mannen T (1981). Circulation of the Spinal Cord. J Neurosurg.

[CR18] Marinlovic SM, Gibo H, Brigante L et al (1997) Arterial blood supply of the spinal cord, and Vertebral artery. Arteries of the Brain and Spinal Cord. Anatomic Features and Clinical Significance, De Angelis Editore, Avellino, Italy:23–48

[CR19] Miyadi T (1931). Pri la angioj de la spinalmedolo ĉe japanoj. I. Pri la arterioj de la spinalmedolo. J Tokyo Med Univ.

[CR20] Nedeltchev K, Loher TJ, Stepper F (2004). Long-term outcome of acute spinal cord ischemia syndrome. Stroke.

[CR21] Novy J, Carruzzo A, Maeder P (2006). Spinal cord infarction clinical and imaging patterns, pathogenesis, and outcomes in 27 patients. Arch Neurol.

[CR22] Sandson TA, Friedman JH (1989). Spinal cord infarction. Report of 8 cases and review of the literature. Medline (Baltimore).

[CR23] Suh TH, Alexander L (1939). Vascular system of the human spinal cord. Arch Neurol Psychiat.

[CR24] Teranishi Y, Kitaoka K, Dan J (2017). Six cases of spinal cord infarction. J C-S Orthop Assoc.

[CR25] Thron AK (1998). Vascular Anatomy of the Spinal Cord. Neuroradiological Investigations and Clinical Syndromes.

[CR26] Watannabe A, Okubo T (2008). Normal anatomy. Jpn J Med Imaging.

[CR27] Yan J, Kanzawa J, Numata N, Jiro H (2016). The right-sided aortic arch with unusual course of bilateral recurrent laryngeal nerves: a report of rare variations. Surg Radiol Anat.

[CR28] Yoshioka K, Tanaka R (2009). CT Angiography and MR angiography of the artery of Adamkiewicz. J Jpn Coll Angiol.

