# Supplementary material for: Differences in distribution of anterior segmental medullary arteries in the cervical and thoracolumbar spinal cord: the “inseln” were characteristics in the cervical spinal cord
Source: Anat Sci Int. 2019 Aug 9;95(1):97–103. doi: 10.1007/s12565-019-00498-y (PMC6942566; doi:10.1007/s12565-019-00498-y)
Supplement: Supplementary file 1 — Supplementary file1 (PPTX 104 kb) [file 12565_2019_498_MOESM1_ESM.pptx]

## Slide 1
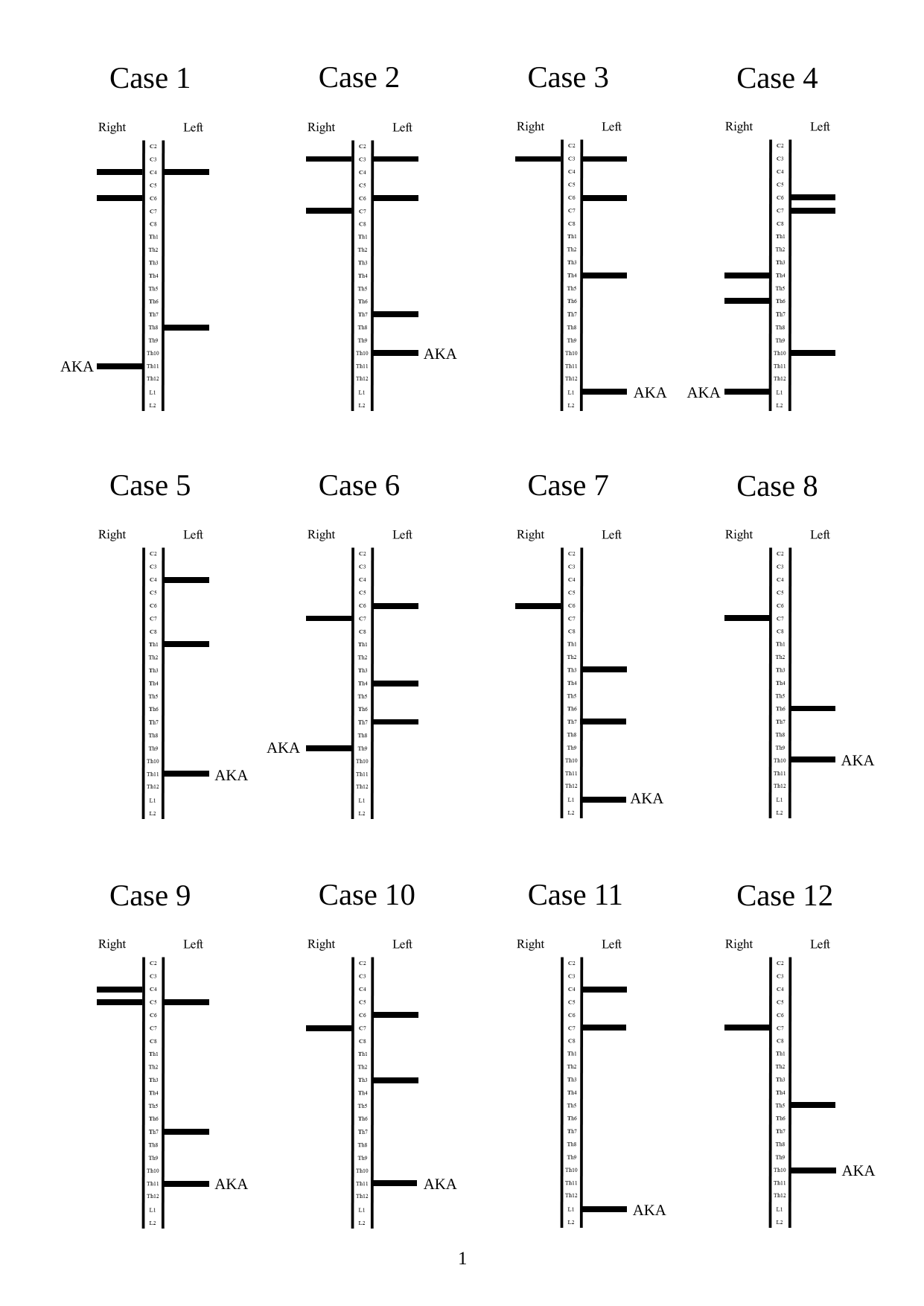

Case 2
Case 3
Case 1
Case 4
AKA
AKA
AKA
AKA
Case 6
Case 7
Case 5
Case 8
AKA
AKA
AKA
AKA
Case 10
Case 11
Case 9
Case 12
AKA
AKA
AKA
AKA
1

## Slide 2
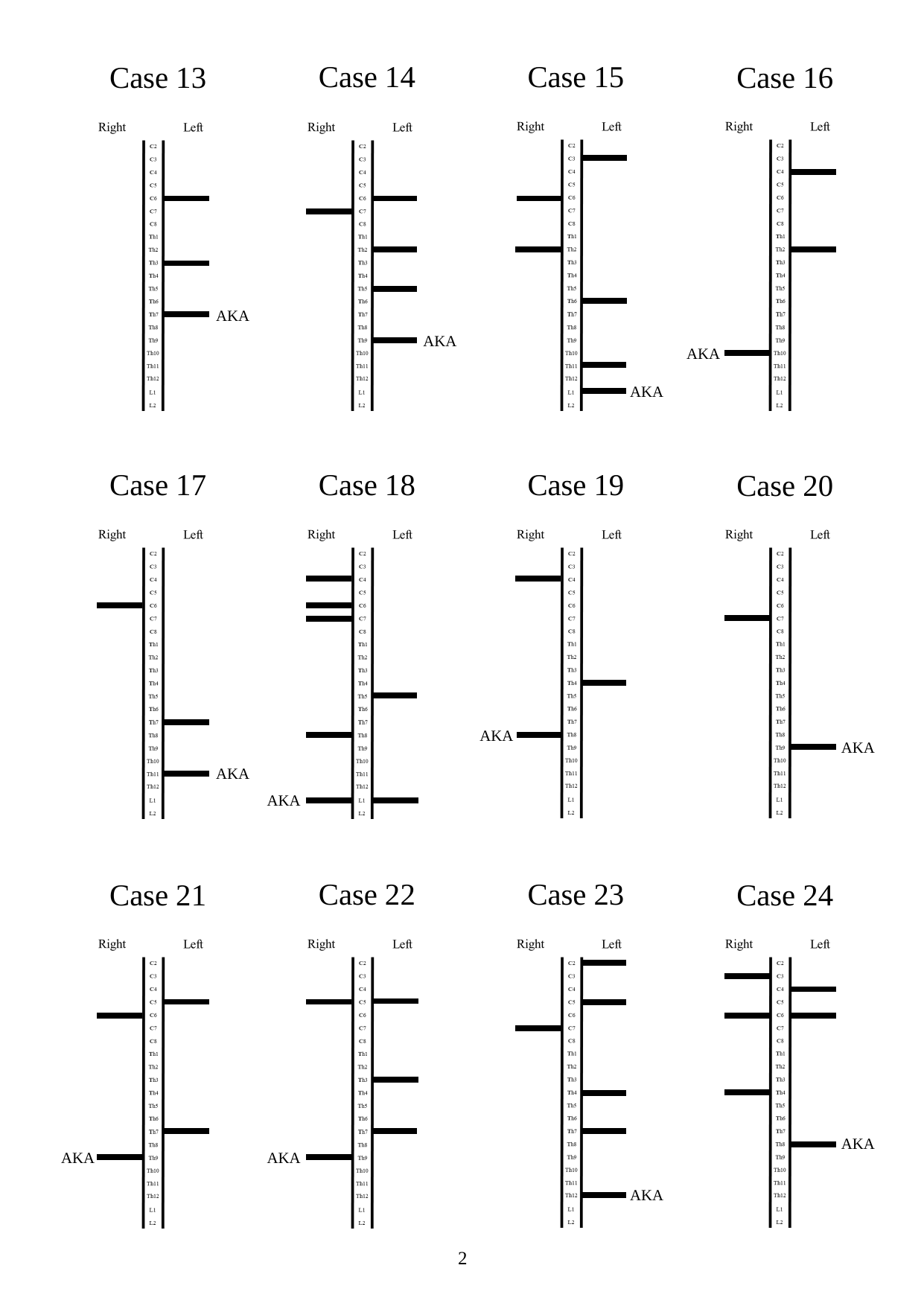

Case 14
Case 15
Case 13
Case 16
AKA
AKA
AKA
AKA
Case 18
Case 19
Case 17
Case 20
AKA
AKA
AKA
AKA
Case 22
Case 23
Case 21
Case 24
AKA
AKA
AKA
AKA
2

## Slide 3
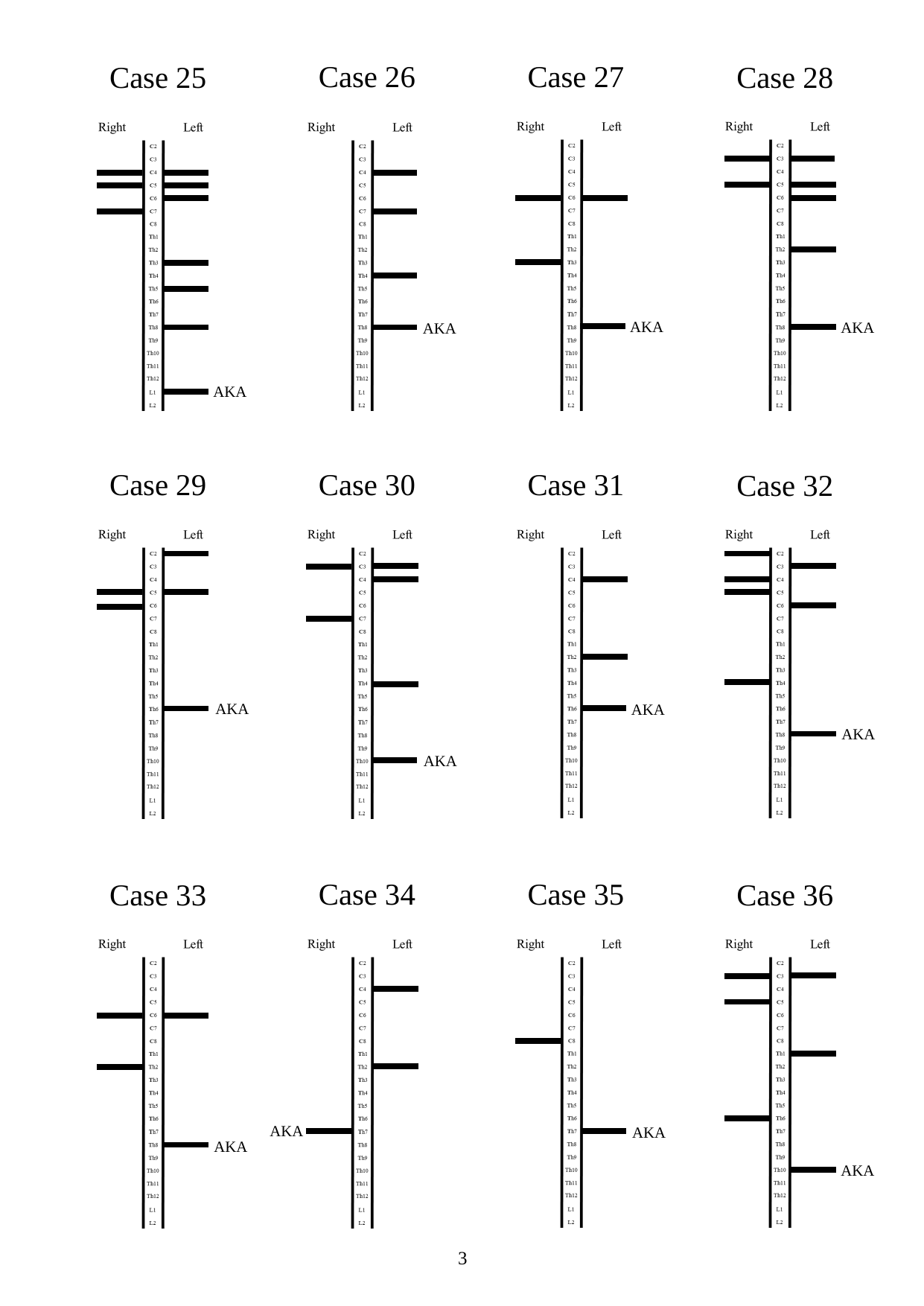

Case 26
Case 27
Case 25
Case 28
AKA
AKA
AKA
AKA
Case 30
Case 31
Case 29
Case 32
AKA
AKA
AKA
AKA
Case 34
Case 35
Case 33
Case 36
AKA
AKA
AKA
AKA
3

## Slide 4
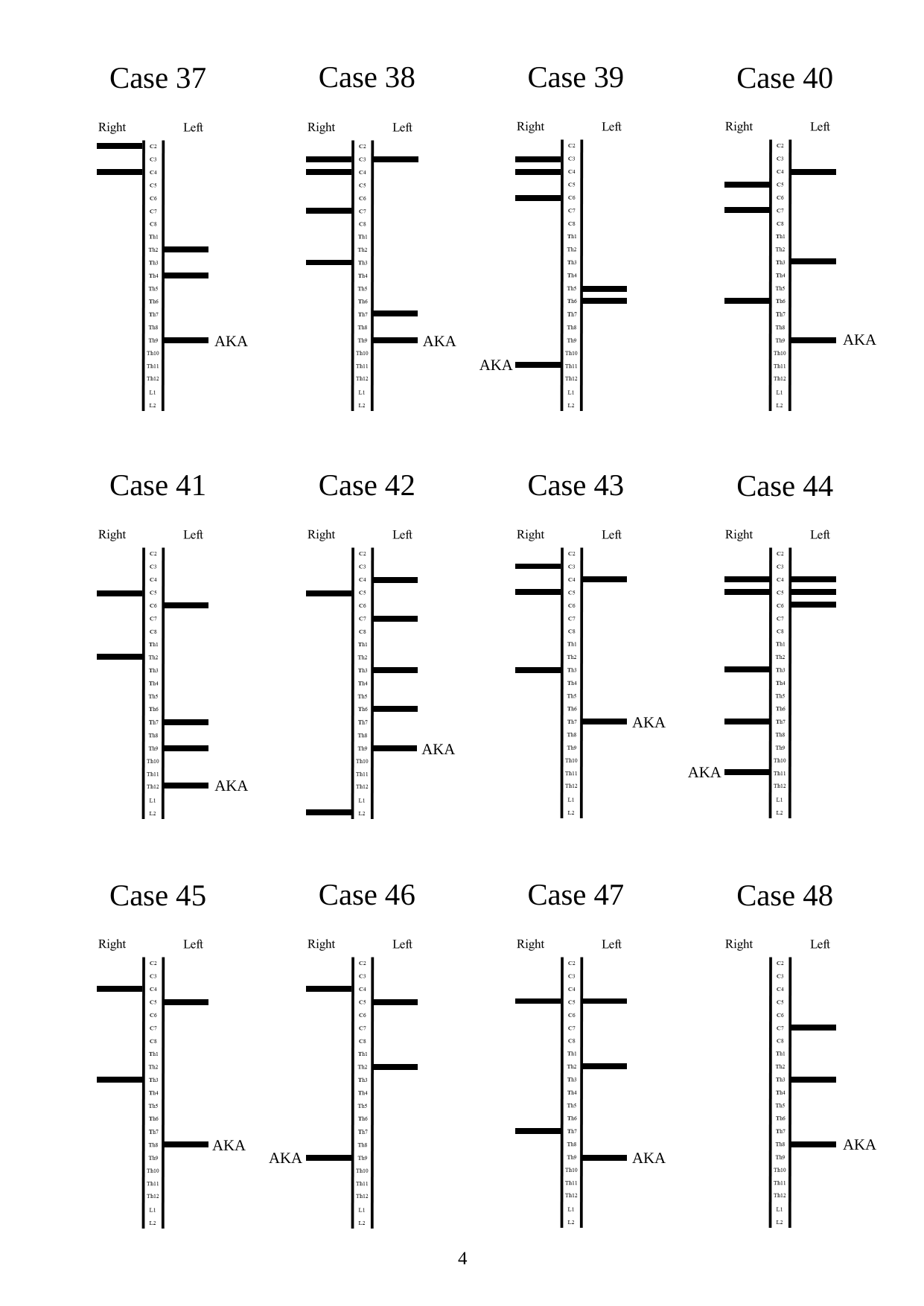

Case 38
Case 39
Case 37
Case 40
AKA
AKA
AKA
AKA
Case 42
Case 43
Case 41
Case 44
AKA
AKA
AKA
AKA
Case 46
Case 47
Case 45
Case 48
AKA
AKA
AKA
AKA
4

## Slide 5
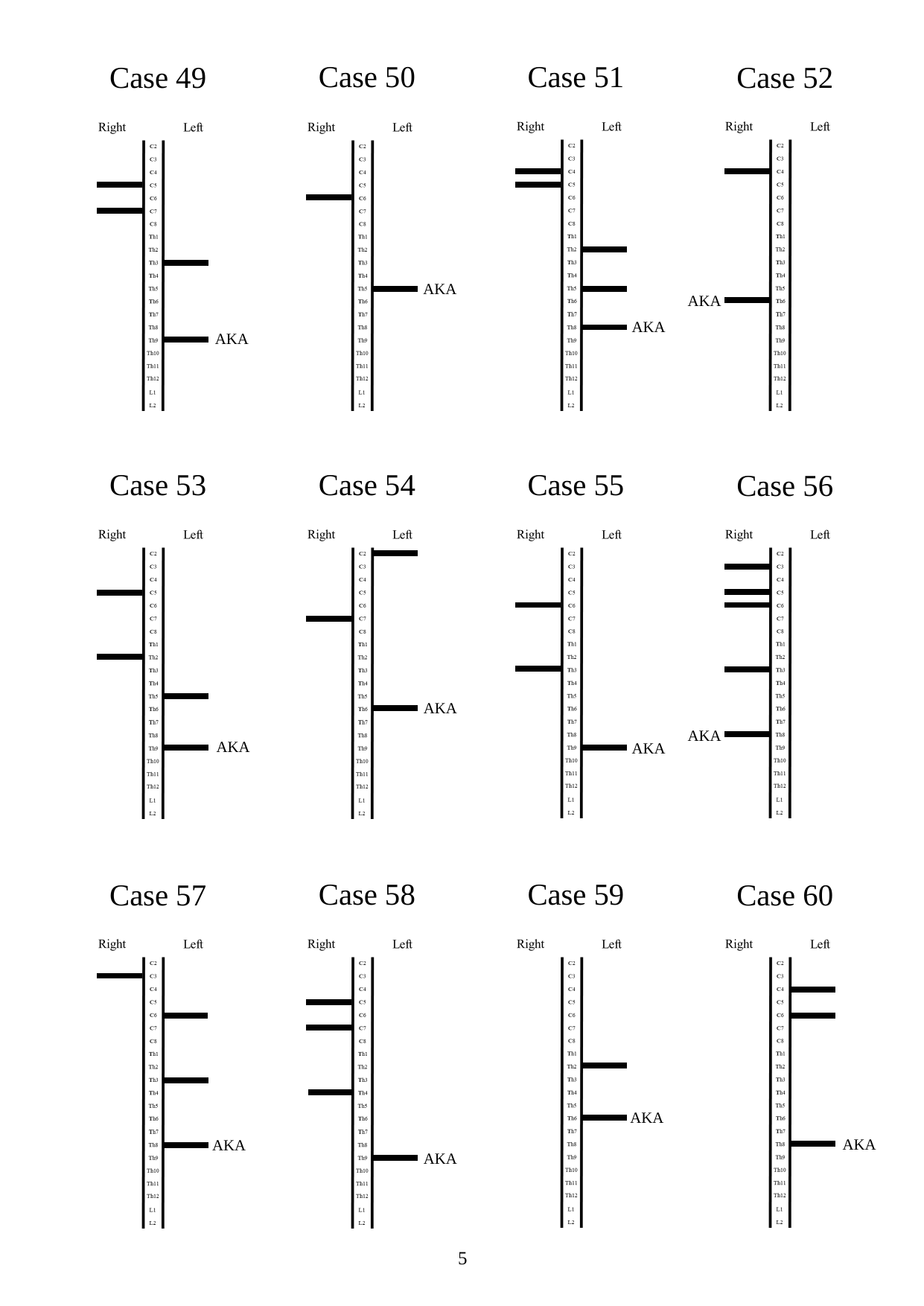

Case 50
Case 51
Case 49
Case 52
AKA
AKA
AKA
AKA
Case 54
Case 55
Case 53
Case 56
AKA
AKA
AKA
AKA
Case 58
Case 59
Case 57
Case 60
AKA
AKA
AKA
AKA
5

## Slide 6
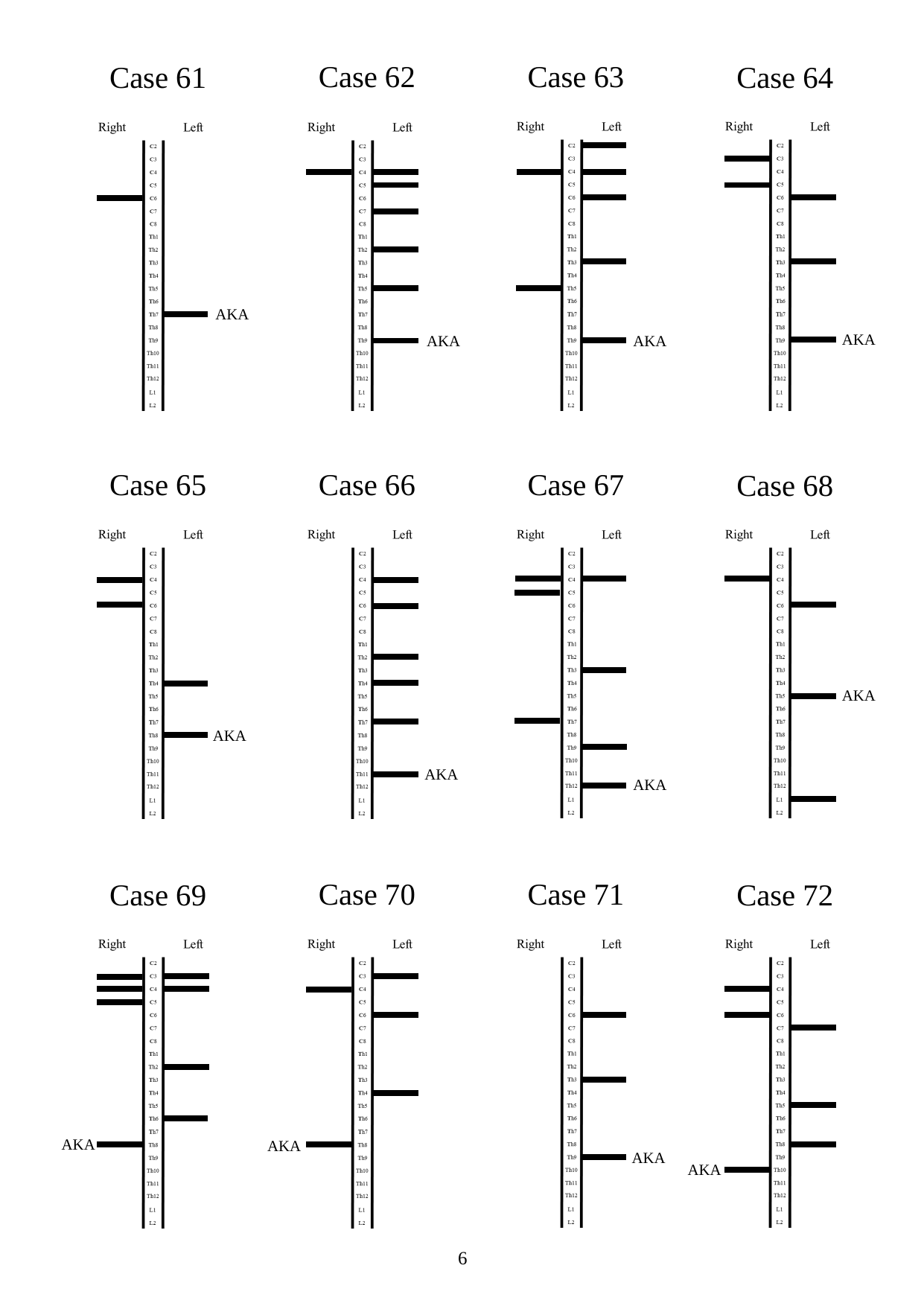

Case 62
Case 63
Case 61
Case 64
AKA
AKA
AKA
AKA
Case 66
Case 67
Case 65
Case 68
AKA
AKA
AKA
AKA
Case 70
Case 71
Case 69
Case 72
AKA
AKA
AKA
AKA
6

## Slide 7
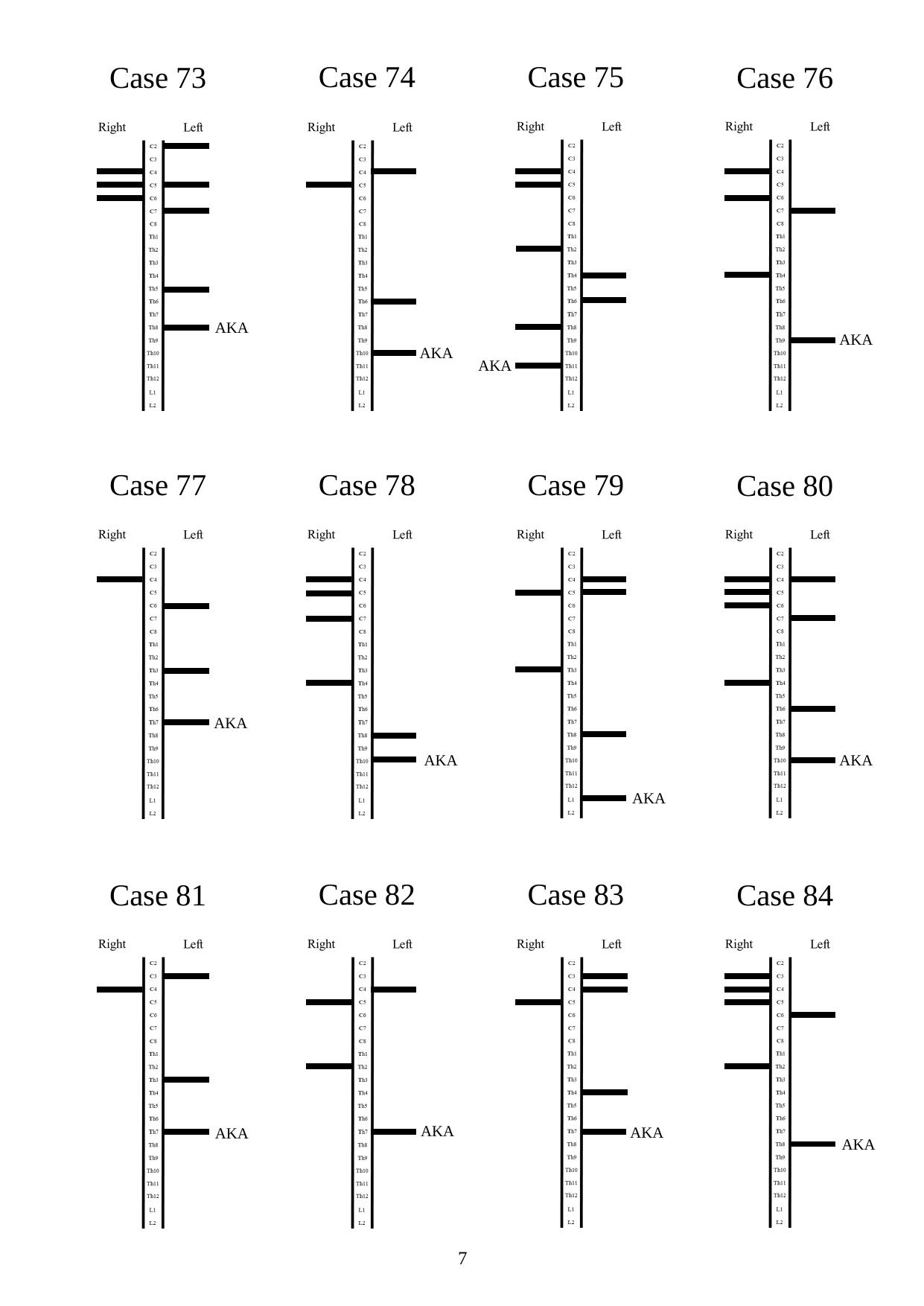

Case 74
Case 75
Case 73
Case 76
AKA
AKA
AKA
AKA
Case 78
Case 79
Case 77
Case 80
AKA
AKA
AKA
AKA
Case 82
Case 83
Case 81
Case 84
AKA
AKA
AKA
AKA
7

## Slide 8
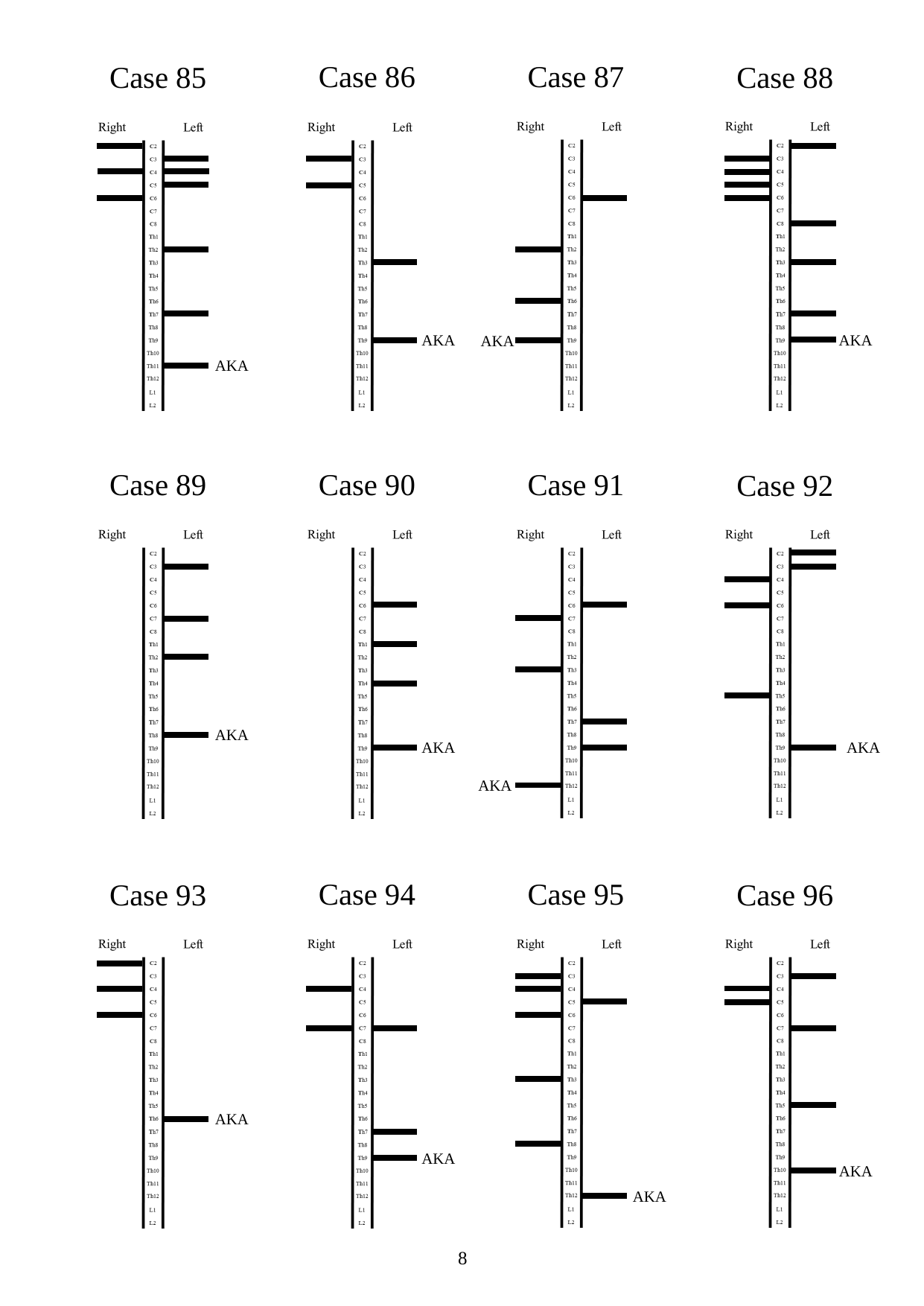

Case 86
Case 87
Case 85
Case 88
AKA
AKA
AKA
AKA
Case 90
Case 91
Case 89
Case 92
AKA
AKA
AKA
AKA
Case 94
Case 95
Case 93
Case 96
AKA
AKA
AKA
AKA
8

## Slide 9
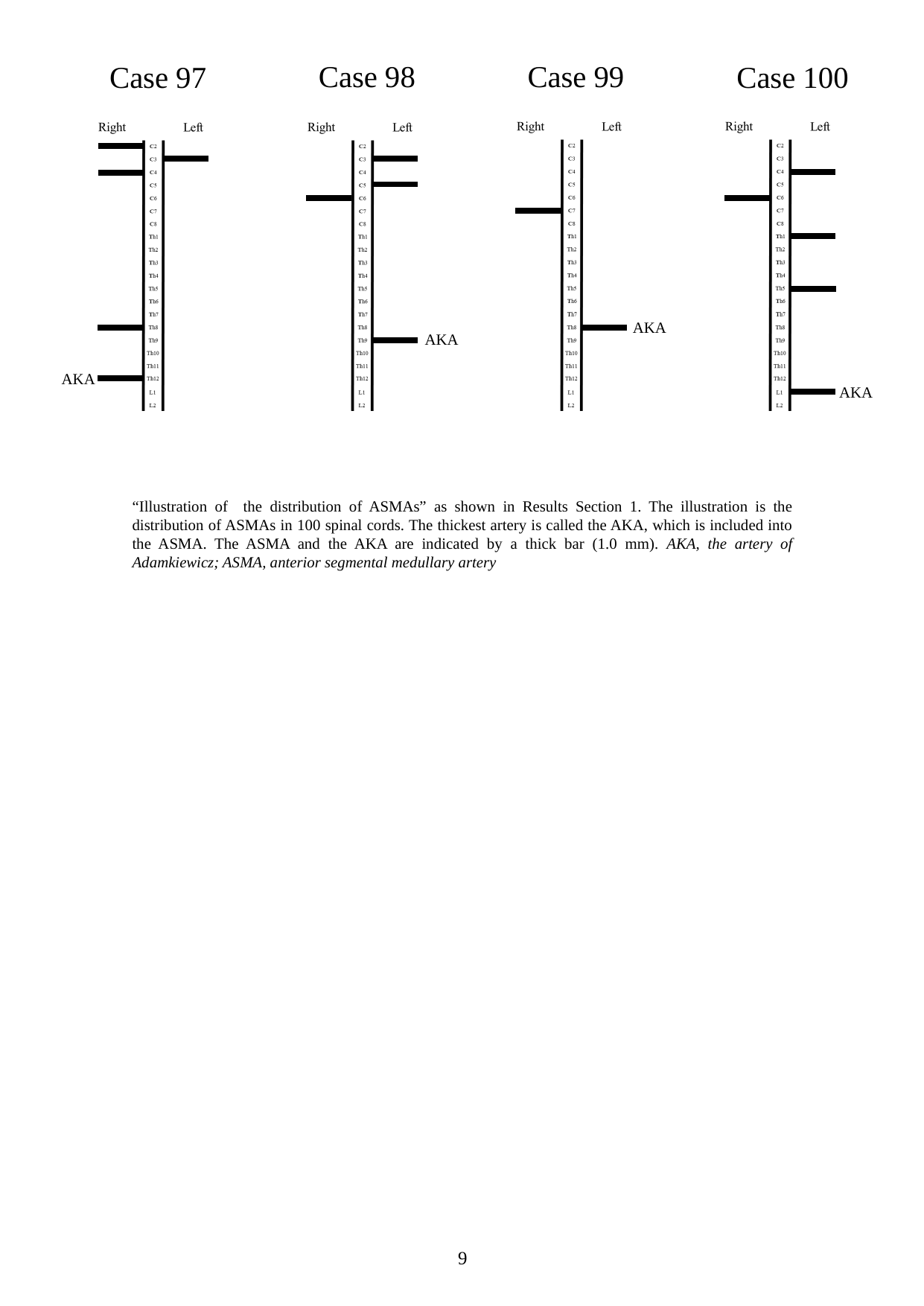

Case 98
Case 99
Case 97
Case 100
AKA
AKA
AKA
AKA
“Illustration of the distribution of ASMAs” as shown in Results Section 1. The illustration is the distribution of ASMAs in 100 spinal cords. The thickest artery is called the AKA, which is included into the ASMA. The ASMA and the AKA are indicated by a thick bar (1.0 mm). AKA, the artery of Adamkiewicz; ASMA, anterior segmental medullary artery
9

## Slide 10
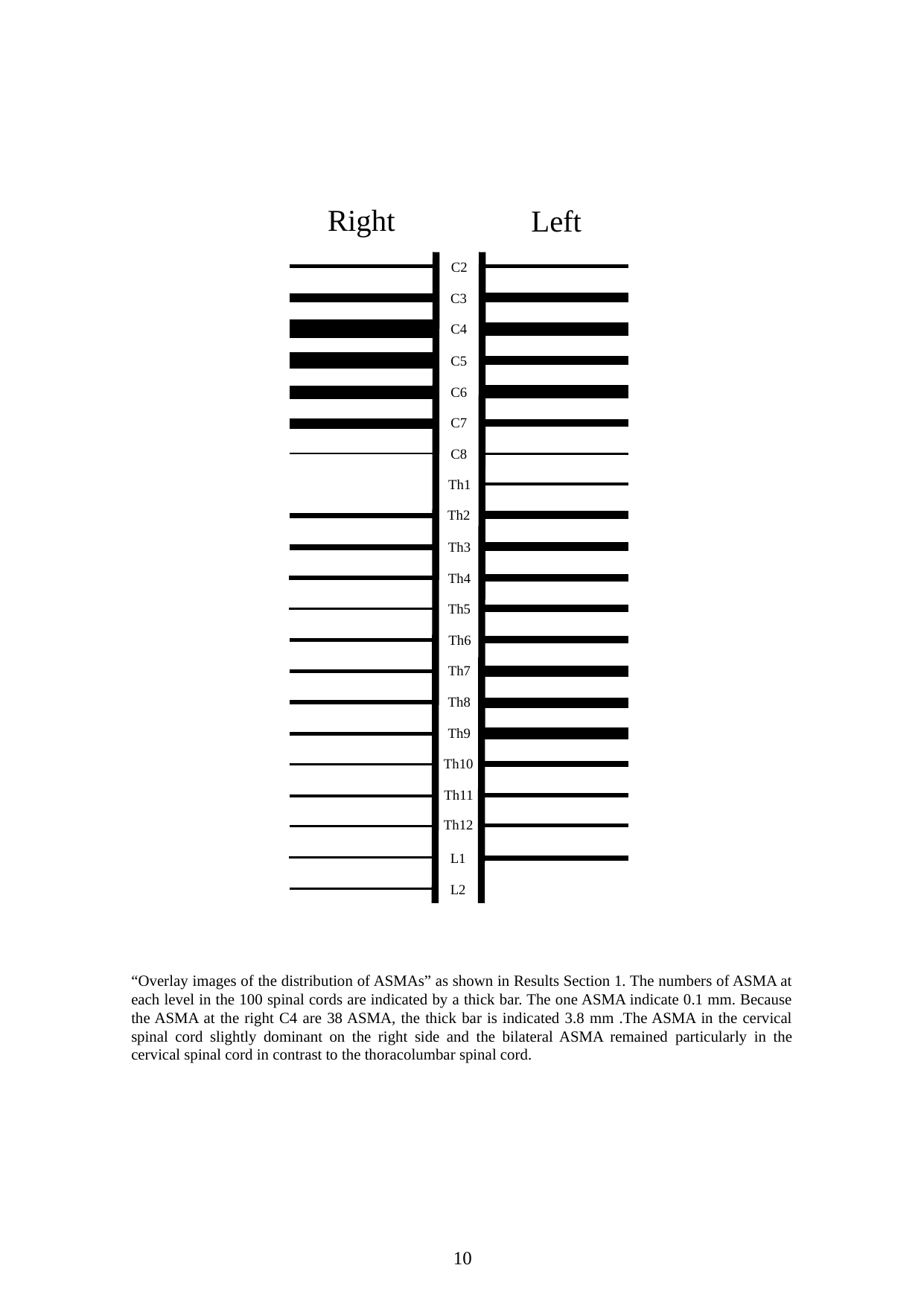

Right
Left
C2
C3
C4
C5
C6
C7
C8
Th1
Th2
Th3
Th4
Th5
Th6
Th7
Th8
Th9
Th10
Th11
Th12
L1
L2
“Overlay images of the distribution of ASMAs” as shown in Results Section 1. The numbers of ASMA at each level in the 100 spinal cords are indicated by a thick bar. The one ASMA indicate 0.1 mm. Because the ASMA at the right C4 are 38 ASMA, the thick bar is indicated 3.8 mm .The ASMA in the cervical spinal cord slightly dominant on the right side and the bilateral ASMA remained particularly in the cervical spinal cord in contrast to the thoracolumbar spinal cord.
10
